# Influences of dietary soy isoflavones on metabolism but not nociception and stress hormone responses in ovariectomized female rats

**DOI:** 10.1186/1477-7827-3-58

**Published:** 2005-10-26

**Authors:** Lihong Bu, Kenneth DR Setchell, Edwin D Lephart

**Affiliations:** 1 Physiology and Developmental Biology Department and Neuroscience Center, Brigham Young University, Provo, Utah, 84602, USA; 2 Department of Pediatrics, Children's Hospital Medical Center, Cincinnati, OH, 45229, USA

## Abstract

**Background:**

Isoflavones, the most abundant phytoestrogens in soy foods, are structurally similar to 17beta-estradiol. Few studies have examined the nociception and stress hormone responses after consumption of soy isoflavones.

**Methods:**

In this study, ovariectomized (OVX) female Long-Evans rats were fed either an isoflavone-rich diet (Phyto-600) or an isoflavone-free diet (Phyto-free). We examined the effects of soy isoflavones on metabolism by measuring body weights, food/water intake, adipose tissue weights as well as serum leptin levels. Also, circulating isoflavone levels were quantified. During chemically induced estrous, nociceptive thresholds were recorded. Then, the animals were subjected to a stressor and stress hormone levels were quantified.

**Results:**

Body weights were significantly lower in Phyto-600 fed rats compared to Phyto-free values within one week and during long-term consumption of soy isoflavones. Correspondingly, Phyto-600 fed animals displayed significantly less adipose deposition and lower serum leptin levels than Phyto-free values. However, rats on the Phyto-600 diet displayed greater food/water intake compared to Phyto-free levels. No changes in thermal pain threshold or stress hormone levels (ACTH and corticosterone) were observed after activation of the hypothalamic-pituitary-adrenal (HPA) stress axis.

**Conclusion:**

In summary, these data show that consumption of soy isoflavones 1) increases metabolism, demonstrated by significantly decreased body weights, adipose tissue deposition and leptin levels, but 2) does not alter nociception or stress hormone responses, as indexed by thermal pain threshold, serum corticosterone and ACTH levels in chemically-induced estrous OVX rats.

## Background

Phytoestrogens are naturally occurring, plant derived, non-steroid molecules that are structurally similar to 17beta-estradiol [1]. Of all the phytoestrogens, soy-derived isoflavones are the most abundant in rodent and human diets and the most studied in both animal and clinical research. Dietary soy isoflavones exist as active aglycones (daidzein and genistein) and inactive glucosides (mainly daidzin and genistin). When consumed, glucosides are hydrolyzed by intestinal glucosidases, which release the aglycones, daidzein and genistein. Daidzein can be further metabolized to a potent and abundant molecule in rodents, equol [2]. The structural similarity between isoflavones and 17beta-estradiol enables isoflavones to exert moderate estrogenic or antiestrogenic properties via mammalian estrogen receptors (ER). It is well established that genistein has a greater affinity for ER beta than ER alpha [3]. Recently, equol has been reported to be anti-androgenic by blocking 5 alpha-dihydrotestosterone (DHT) in the circulation and presumably within cells [4]. Moreover, equol appears to bind ER beta > ER alpha, similar to that of genistein [2]. The influences of equol are likely to be substantial due to the high level of equol (1,000~2,500 ng/ml) compared to 17beta-estradiol (10~100 pg/ml) in the circulation of rodents [4], that consume a soy-based diet.

Of all the studies examining the effects of soy isoflavones, most investigative attention has been on age-related diseases and hormone-dependent cancers [1,3,5-9]. However, few studies have examined the influence of soy isoflavones on metabolism, nociception and stress hormone responses. It is known from our previous studies that dietary soy isoflavones significantly increase food/water intake while at the same time significantly decreasing body weight in intact male and female rats [4,10]. In reference to pain thresholds, in one study, dietary soy consumption suppressed neuropathic pain in rats after partial sciatic nerve ligation [11]. Also, it has been reported that genistein and daidzein decreased cortisol synthesis by suppressing the activity of P450c21 in cultured adrenal cortical cells [12]. Phytoestrogens have received increased research interest as an alternative to estrogen replacement therapy due to their selective estrogen receptor modulator (SERM)-activity [1]. However, effects of phytoestrogens on nociception and the hypothalamic-pituitary-adrenal (HPA) stress axis during steroid replacement therapy have not been investigated *in vivo *in previous studies. In this study, we used OVX Long-Evans rats to mimic the surgically postmenopausal condition and studied the effects of dietary soy isoflavones on metabolism by measuring body weight, food/water intake, adipose tissue deposition as well as serum leptin levels. Then, steroid replacement therapy was reproduced in OVX rats with the administration of a steroid regimen (estrogen then progesterone) to induce an LH surge and estrus. Nociception and stress hormone responses were studied by examining thermal pain thresholds, serum corticosterone and ACTH levels during the chemically-induced estrous state.

## Methods

### Animals

OVX Long-Evans female rats at 50 days old were purchased from Charles River Laboratories (Wilmington, MA, USA). These animals were ovariectomized prior to shipping. They were caged individually and housed on a light/dark schedule (lights on 0600–1900 h) in the Brigham Young University Bio-Ag vivarium. The animals and methods of this study were approved by the Institute of Animal Care and Use Committee (IACUC) at Brigham Young University.

### Treatment-Diets

The diet from the supplier (Ziegler Bros., NIH07, Gardner, PA, USA) contained approximately 200 ppm isoflavones. Upon arrival the animals were allowed ad libitium access to water and either a commercially available diet with high isoflavone levels (Harlan Teklad Rodent Diet 8604, Madison, WI, USA) containing approximately 600 ppm of soy isoflavones (referred to hereafter as the Phyto-600 diet), or a custom diet (Ziegler Bros., Sterol free diet, Gardner, PA, USA) containing approximately 10–15 ppm of soy isoflavones (referred to hereafter as the Phyto-free diet) [13,14]. The composition of these diets is described in detail elsewhere [14]. The diets were balanced and matched for equivalent percentage content of protein, carbohydrate, fat, amino acids, vitamins and minerals, etc. Circulating serum isoflavone levels from rats maintained on these diets have been reported previously by our laboratory using GC/MS analysis [14] and are reported here for OVX rats. All the rats were sacrificed at 94 days of age after approximately 6 weeks on the diet treatments.

### Weight Measurements

Body weights were measured on a Metter 1200 balance (St. Louis, MO, USA) at 50, 58 and 93 days of age, when the animals were delivered, one week and six weeks after consuming the treatment diets, respectively. White (in the abdominopelvic cavity surrounding reproductive tissues) and brown adipose (inter-scapular) tissues were dissected and weighed on a Sartorious balance (Brinkman Inst. Co., Westbury, NY, USA). Food intake was measured on a Metter 1200 balance and water intake was measured in drinking tubes for three consecutive days at the age of 85 days. For each animal, the average food and water intake per day was calculated.

### Pain Threshold Level and Stress Response

Two days before pain threshold was measured, all OVX rats received a subcutaneous injection of 0.1 mg estradiol benzoate (EB) in 0.1 ml oil at 1200 h. A subcutaneous injection of 1.0 mg progesterone in 0.1 ml oil was administered 42 hours after the EB injection [15,16]. Six hours after the progesterone injection, pain threshold levels were measured (during 1200~1230 h or during the steroid-induced LH surge) [17] using a hot-plate analgesic meter (Columbus Instruments, Columbus, Ohio, USA). Serum was collected at 40 hours after EB injection and 6 hours after the progesterone injection for the detection of LH levels with a kit from Diagnostic Systems Laboratories (Webster, Texas, USA). After the body weight of each animal was measured, one animal was placed on the hot-plate apparatus (uniform plate temperature was set at 50°C) and the time in seconds was recorded until the animal licked its back paw (latency to paw lick). Immediately following the hot-plate test, each animal was subjected to restraint, illumination and heat stress for 5 minutes, as previously performed in our laboratory [18]. In general, the animals were placed in a 150 mm (l) × 60 mm (w) × 40 mm (h) Plexiglas tube. The tube was under the illumination of two 150-W flood-lamps (generating 2200 lm/m^2^) and the intratube temperature reached 31–34°C during the stress interval.

### Stress Hormones and Leptin RIA

Immediately after the exposure to the stress protocol (above), each rat was sacrificed, trunk blood collected, serum prepared and stored at -20°C until assayed for hormone levels. Serum corticosterone levels were assayed with a kit from Diagnostic Products Corporation (Los Angeles, CA, USA). Serum ACTH concentrations were quantified with a kit from Diagnostic Systems Laboratories (Webster, Texas, USA). Serum leptin levels were determined by a kit from Linco Res. Inc. (St. Charles, MO, USA). All samples were run in duplicate in the respective assays with internal control samples and the intra-assay coefficients of variance were 7% (corticosterone), 9% (ACTH) and 10% (leptin).

### Statistical Analysis

All the data were expressed as Mean ± SEM and analyzed by the Statistical Analysis System (SAS). The data were tested by the two-sample Student's t-test, or where appropriate by repeated measures based mixed-model analysis and considered significantly different at p < 0.05.

## Results

### Circulating serum isoflavones levels by diet treatment

**Figure 1:**
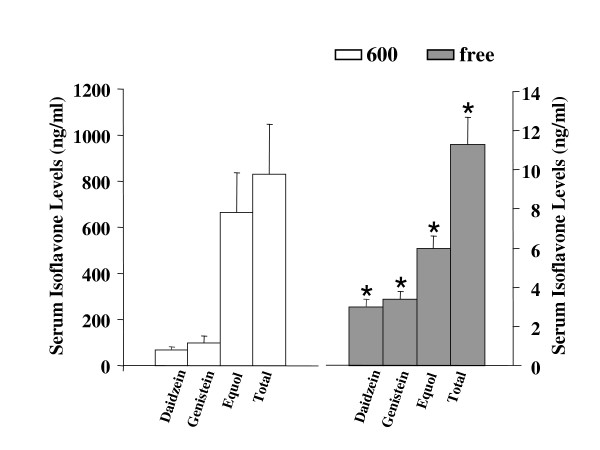
Circulating serum soy isoflavone levels of 94-day-old OVX Long-Evans rats in ng/ml fed either an isoflavone-rich (600) or an isoflavone-free (free) diet. The analysis was performed by GC/MS. The Phyto-free fed animals displayed significantly lower daidzein, genistein, equol, or total isoflavone levels compared to the Phyto-600 values (*).

Figure 1 illustrates the levels and types of isoflavones from 94 day-old OVX rats fed the Phyto-600 versus the Phyto-free diet from 50 days of age. The rats fed on the Phyto-600 diet displayed significantly higher levels of daidzein, genistein, equol (a known metabolite of daidzein) and total isoflavone compared to animals on the Phyto-free diet. Equol was the major circulating isoflavones, whereas, daidzein and genistein represented lower percentages of the total circulating isoflavones.

### Effects on metabolism of dietary soy isoflavones

**Figure 2:**
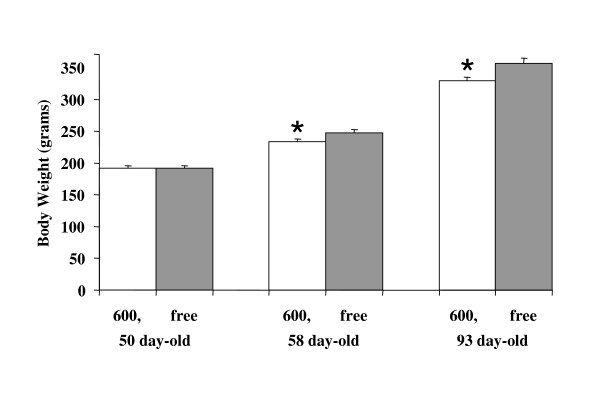
Effects of dietary soy isoflavones on body weight in OVX Long-Evans rats, fed either an isoflavone-rich (600) or an isoflavone-free (free) diet. At 58 and 93 days old, 600 body weights (*) were significantly lower compared to free fed OVX rats.

**Figure 3:**
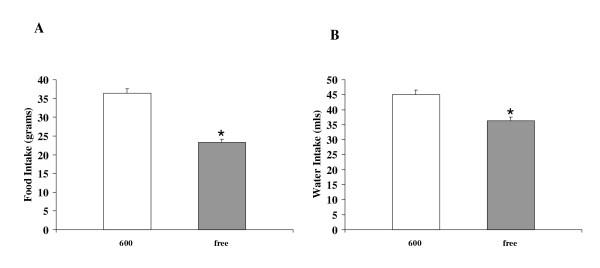
Effects of dietary soy isoflavones on average food and water intake per day in 85 day-old OVX Female Long-Evans Rats. Rats fed an isoflavone-rich (600) diet displayed significantly less (*) food (A) and water intake (B) compared to rats fed an isoflavone-free (free) diet.

Body weights were significantly lower in Phyto-600 fed OVX rats compared to Phyto-free group within one week and after long-term (approximately six week) consumption of soy isoflavones [Figure 2]. Interestingly, the Phyto-600 fed OVX rats displayed significantly higher food and water intake (36.3 ± 1.2 g and 45.0 ± 1.6 ml) compared to the Phyto-free fed animals (23.3 ± 0.8 g and 36.3 ± 1.2 ml) at approximately 85 days of age [Figure 3].

**Figure 4:**
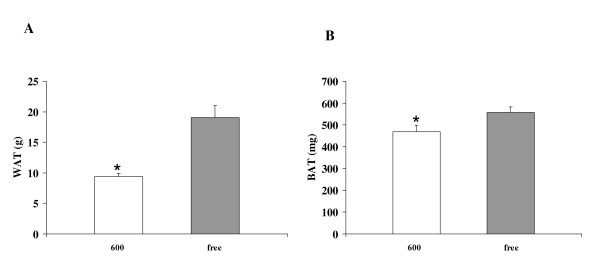
Effects of dietary soy isoflavones on white adipose tissue (WAT) and brown adipose tissue (BAT) weights from 94 day-old OVX female Long-Evans rats. White adipose tissue and brown adipose tissue weights were significantly lower in rats fed the 600 diet (*) compared to the free fed animals.

**Figure 5:**
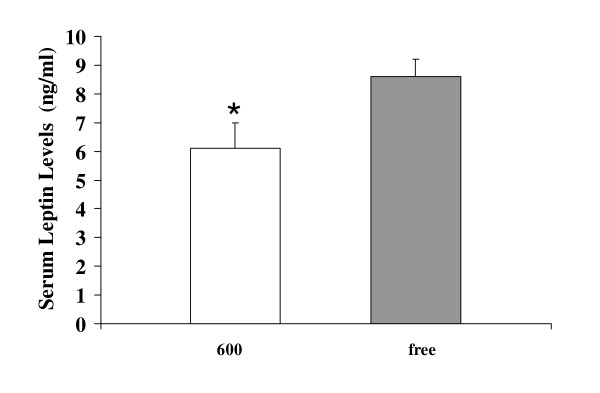
Effects of dietary soy isoflavones on serum leptin levels. The 600 fed animals displayed significantly lower leptin levels (*) compared to the free fed animals, a finding consistent with the white adipose tissue weight results (by diet treatment) shown in figure 4.

Exposure to dietary soy isoflavones significantly decreased white and brown adipose tissue weights of OVX rats [Figure 4]. White adipose tissue deposition in the Phyto-600 rats (9.4 ± 0.5 g) was about 50% less than that in the Phyto-free group (19.1 ± 1.9 g). Correspondingly, serum leptin levels of Phyto-600 fed OVX rats were significantly lower compared to Phyto-free values [6.1 ± 0.9 vs. 8.6 ± 0.6, ng/ml ± SEM, Figure 5].

### Pain threshold

**Figure 6:**
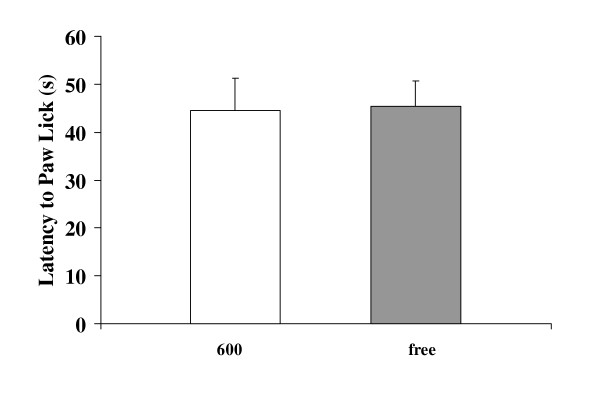
Effects of dietary soy isoflavones on nociception. The 600 fed animals displayed similar thermal pain threshold, latencies to paw lick [in seconds (s)] on the hot plate, to the free fed animals.

To evaluate the effects of dietary soy isoflavones on nociception during the sequential injection of steroid-induced LH surge, the thermal pain threshold was measured as the latency to paw lick (time in seconds on the 50°C hot plate until licking the back paw). The LH surge was confirmed to be induced with sequentially injection of EB and progesterone by measuring the LH levels in serum [40 hours after EB injection: Phyto-600 values = 4.7 ± 0.8 vs. Phyto-free values = 4.5 ± 0.4; 6 hours after progesterone injection: Phyto-600 values = 21.1 ± 1.4 vs. Phyto-free values = 21.9 ± 1.6; ng/ml ± SEM; data not shown graphically]. No significant difference was observed between the diet treatment groups [Phyto-600 values = 44.5 ± 6.7 vs. Phyto-free values = 45.4 ± 5.3; second ± SEM, Figure 6] for the pain threshold parameter (latency to paw lick).

### Stress hormones

**Figure 7:**
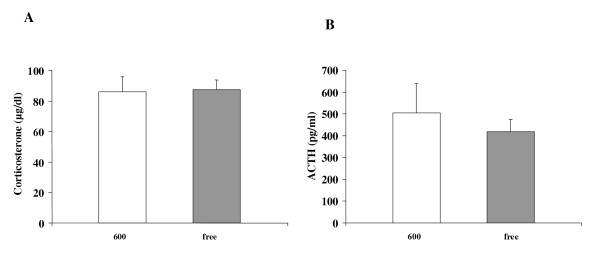
Effects of dietary soy isoflavones on stress hormones from 94 day-old OVX Long-Evans rats after activation of the hypothalamic-pituitary-adrenal (HPA) stress axis during chemically induced estrus. No significant changes on corticosterone (A) or ACTH (B) were observed between the Phyto-600 and Phyto-free group.

Immediately after the pain threshold test, the animals were restrained for 5 minutes (see methods above) and the stress hormones (corticosterone and ACTH) were determined from serum samples, collected immediately after the stress interval. No significant changes of either corticosterone [Phyto-600 values = 85.8 ± 10.0 vs. Phyto-free values = 87.3 ± 6.5; μg/dl ± SEM; Figure 7] or ACTH [Phyto-600 values = 505 ± 134 vs. Phyto-free values = 418 ± 58; pg/ml ± SEM; Figure 7] were detected with the consumption of soy isoflavones during the chemically induced LH surge.

## Discussion

The effects of dietary phytoestrogens on metabolism, nociception and stress response were studied in the postmenopausal condition, mimicked by using 94 day-old OVX Long-Evans rats. The present data showed that consumption of soy isoflavones increases metabolism, demonstrated by significantly decreased body weight, adipose tissue deposition and leptin levels. But during a chemically induced LH surge, dietary soy isoflavones do not alter nociception or stress hormone responses, as indexed by thermal pain threshold, serum corticosterone and ACTH levels.

We have previously reported that a variety of behavioral, metabolic and neuroendocrine parameters are influenced by consumption of soy isoflavones in intact animals [4,10,14]. The increased metabolism observed in this study from OVX rats is in agreement with our previous findings in males, showing significant decreases in body weight, white and brown adipose tissue weight and serum leptin levels [19,20]. Notably, the decrease of body weight in OVX rats, in the present study, was significant within about one week's consumption of the Phyto-600 diet. It is reported that genistein has the ability to increase lipolysis in isolated rat adipocytes and decrease lipogenesis in white adipose tissue in OVX rats [21,22]. This may account, at least in part, for the reduced body weights in Phyto-600 fed OVX animals. Additionally, it has been shown that locomotor activity is increased in Phyto-600 fed animals, along with increases in thyroid hormone levels [14,20]. This may play a role in further reducing body weights, even though the food/water intake of these animals is significantly greater than Phyto-free fed animals, which is in accord with other studies in OVX animals [23]. Since leptin is synthesized and secreted from white adipose tissue, it is not surprising to find the significantly reduced leptin levels in Phyto-600 animals compared to Phyto-free group. Consequently, the higher hypothalamic NPY levels, as a result of negative feedback from lower leptin levels, stimulate feeding behavior [20]. The finding of decreased brown adipose tissue weight is similar to the results from another study in which male rats consumed soy isoflavones [20]. However, the biological effects of this decrease are not clear. Hence, further studies are necessary to determine the mechanisms of how metabolic levels are increased by the consumption of soy isoflavones.

The use of natural remedies is omnipresent in addressing women's health issues, especially in light of the negative risk factors associated with estrogen replacement therapy in postmenopausal women [1,5,24,25]. Some recent studies suggest that soy consumption (especially via dietary supplements) is effective in treating symptoms of peri- and postmenopause [26]. In this regard, OVX rats were administered a steroid regimen (estrogen then progesterone) to induce an LH surge and estrus, in order to reproduce, in part, steroid replacement therapy [27]. It has been shown that responsiveness to noxious stimuli change after gonadal steroid treatment and during the estrous cycle [28]. There is evidence that LHRH may interact with central opioid systems causing an increased sensitivity to nociceptive stimulation (hyperalgesia) and reduction of the antinociceptive effect of morphine in female rats [29]. Thus, using this steroid paradigm in the present study, induced LH surges were stimulated in all the OVX rats and thus one might predict that pain sensitivities would increase. However, no significant difference was observed between Phyto-600 vs. Phyto-free fed OVX animals in the present pain threshold experiment, and our recorded time (paw lick) latencies were within range of previous pain threshold studies, but higher than those recorded in intact male rats (Phyto-600 values = 25.94 ± 3.1 vs. Phyto-free values = 24.14 ± 2.5; second ± SEM) [30]. While prior examination of this parameter is scarce in relation to soy consumption, a recent report did not show a reduction in thermal pain in soy fed animals [11], a finding similar to that of our present results.

It was reported that genistein and daidzein decrease cortisol synthesis by suppressing the activity of P450c21 in cultured adrenal cortical cells [12], our data showed similar post-stress corticosterone levels between Phyto-600 and Phyto-free fed OVX female rats. The lack of detecting any difference in corticosterone levels, in the present *in vivo *study compared to prior *in vitro *results [12], is most likely due to the adrenal cortex being maximally stimulated by the high ACTH levels in both the Phyto-600 and Phyto-free groups [31].

In intact male rats, results obtained after similar stressor, ACTH levels were significantly greater in the Phyto-600 vs. the Phyto-free fed group, signifying that dietary soy isoflavones may increase the hypothalamic-pituitary stress response [30]. The finding of no differences in ACTH levels, in the present OVX rats, suggests that gender differences exist in the biological actions of dietary soy isoflavones, especially in relationship to the stress response where females typically display significantly higher stress hormone levels compared to males [32]. Finally, future time-dependent stress response studies may determine whether consumption of soy isoflavones reduce glucocorticoid release from the adrenal cortex.

## Conclusion

The present *in vivo *study shows that consumption of a commercially available soy-based rodent diet increases metabolism, demonstrated by significantly decreased body weights, adipose tissue deposition and leptin levels in OVX female rats. However, in female rats studied here, nociception and stress hormone responses, as indexed by thermal pain threshold, serum corticosterone and ACTH levels, were not influenced by consumption of soy isoflavones. However, whether the effects are due to soy protein or to differences in isoflavones is unknown. Further research is warranted due to the pervasive biological actions of dietary soy isoflavones altering physiology and behavior.
